# The Book World of Medicine and Science

**Published:** 1895-11-02

**Authors:** 


### The Book World of Medicine and Science.

The Herschels and Modern Astronomy. By Agnes M. Clerke. (The Century Science Series. Cassell and Co. London. 1895.)

Miss Clerke has been fortunate in the choice of subject allotted her in the Century Science Series, and she has, moreover, made good use of hes materials. What is especially striking in this book is the lesson it brings before our minds as to the results of a long and sustained intention—an "intention" on the part of the Herschels which raised them out of complete obscurity, and placed them foremost among the scientists of their day. Take the case of the elder Herschel, for instance. His advantages intellectually were those of an ordinary School Board child—certainly not more. He was brought up as oboeist in his father's regimental band, and the career which he followed till well on in manhood was that of a musician. Until he was thirty-five years of age astronomy was little but a pastime to him. It must be borne in mind that when he came to England, in his nineteenth year, he had nothing but a French crown-piece in his pocket, and that by having come to England, he incurred the penalties of desertion. (These were never exacted, and were remitted in 1782 by George III.). Under these circumstances, the boy had few advantages to recommend him besides an unbounded ambition, a keenness of intellect above his fellows, and a great determination. He soon rose to the foremost ranks in his profession at Bath, where he settled; but a musical career did not satisfy William Herschel. The inner promptings of genius told him to look beyond. The few spare hours which fell to the lot of a popular teacher and composer of music, he diligently devoted in responding to the calls of an "undeclared vocation," as Miss Clerke defines it, and he applied himself in seriously studying mathematics, optics, and finally astronomy. He had no sooner fallen under the spell of this last science, than he resolved to "take nothing upon trust, but to see with his own eyes all that other men had seen before." Such, he explains in his own words, was the spirit which prompted his work. Sir William Herschel was a genius, but something besides, he was the hardest, the most indefatigable of workers. The history of his work, in which his devoted sister Letitia so ably assisted him, reads like a romance. His latest biographer has not failed to seize upon the romantic element of his life; indeed, there is more of the "Herschels" in her book than there is of actual modern astronomy. Miss Clerke sketches in also the life of Sir William's sister, the patient, devoted sharer of her brother's toils, and the third part of the book is devoted to Sir John Herschel, a man of perhaps yet more versatile intellectual capacities than the two preceding him in this volume. A man of great religious conviction and philosophical tendencies he was, on whom also had descended the mantle of his father's genius.

Weather and Disease. By Alex. B. MacDowall. (London: The Graphotone Company. 1895. Pp. 93.)

This is mainly an attempt to place before the reader in graphic form certain facts regarding variations of weather and disease. Not only are tables of figures dull, but they are relatively uninstructive, and there can be no doubt that it is only when they are thrown into graphic curves that many of the lessons which they should teach become apparent. Some of the tables are very striking, as, for example, one showing the relation between the curve of the London death-rate from diarrhœa and dysentery and that of the mean temperature at Greenwich during July since the year 1838, and another showing the relation between the death-rate from respiratory diseases in the first quarters of a series of years and the mean temperatures at the same periods. Very curious also are the relations which are shown to exist between the prevalence of sunspots and various meteorological phenomena.

Cancers and Tumours. Reprinted papers by Herbert Snow, M.D.Lond. (London: J. and A. Churchill. 1895. Pp. 63.)

Dr. Snow has done well to place in collected form the four papers of which this pamphlet consists, for they cover some of the most interesting practical questions in reference to the treatment of cancer. Within the lifetime of the older among us a complete change has taken place in the ideas held regarding the curability of cancer, but along with the growing assurance of the possibility of cure by sufficiently early excision comes a heavier responsibility upon the surgeon, for it is clear that to effect a cure removal must be both early and complete. Great emphasis is laid on the fact that lymph glands are infected long before they are enlarged, and upon the importance of complete removal of all glands which lie in direct lymph track whether enlarged or not. The moral constantly insisted on is the necessity of early diagnosis and prompt action. These articles should be carefully read by general practitioners, under whose care the earlier stages of such cases commonly come, for if Dr. Snow is right in his conclusion, anyone who is not prepared himself to operate should at once pass on all cases of tumour to those who will undertake that responsibility, for it is to be feared that people who suffer from tumours are only too often advised to wait to see how they go on, which in the case of cancer is fatal.

